# Toward Sex-Specific Biomaterials Innovation: A Perspective

**DOI:** 10.1021/acsbiomaterials.5c00342

**Published:** 2025-08-20

**Authors:** Gerry L. Koons

**Affiliations:** † Regenerative Biomaterials Research Group, Radboud University Medical Center, Nijmegen 6525 GA, The Netherlands; ‡ Computational Pathology Research Group, Research Institute for Medical Innovation, Radboud University Medical Center, Nijmegen 6525 GA, The Netherlands

**Keywords:** precision biomaterials, hormonal differences, women’s health, regenerative medicine

## Abstract

Sex-related differences influence key biological processes relevant to biomaterials research, including tissue regeneration, immune response, drug metabolism, and relevant diseases. Despite increasing recognition of sex as a critical biological variable, biomaterials research has historically relied on generalized study designs that fail to account for sex-specific physiological variability. This oversight has contributed to inconsistencies in biomaterial performance, reduced predictive accuracy in preclinical studies, and increased the risk of suboptimal clinical outcomes. A transition toward sex-specific biomaterials research is necessary to advance precision medicine and ensure that biomaterials are designed to perform optimally across different patient populations. This perspective examines the motivations and barriers to integrating sex-related differences in biomaterials, including current approaches to (pre)­clinical study design, data generation and sharing, funding realities, and regulatory frameworks. This work also highlights biomaterials applications for female-specific conditions, from pelvic floor disorders and uterine wound healing to endometriosis. Furthermore, biomaterials applications for diseases such as osteoporosis, diabetes, and cancer are discussed in terms of sex-related differences in immune response, drug metabolism, and tissue regeneration and the subsequent impact on biomaterial performance. To address these challenges, this perspective proposes strategies for improving biomaterials research, including the adoption of standardized experimental frameworks, integration and aggregation of sex-specific analyses in data sets, and application of artificial intelligence-driven biomaterial evaluation. Priorities for collaborative partners, funding bodies, and regulatory agencies in facilitating these solutions are outlined to improve scientific practice and clinical applicability. By implementing these strategies, biomaterial designs can move beyond a one-size-fits-all paradigm and improve alignment with the principles of precision medicine.

## Graphical abstract

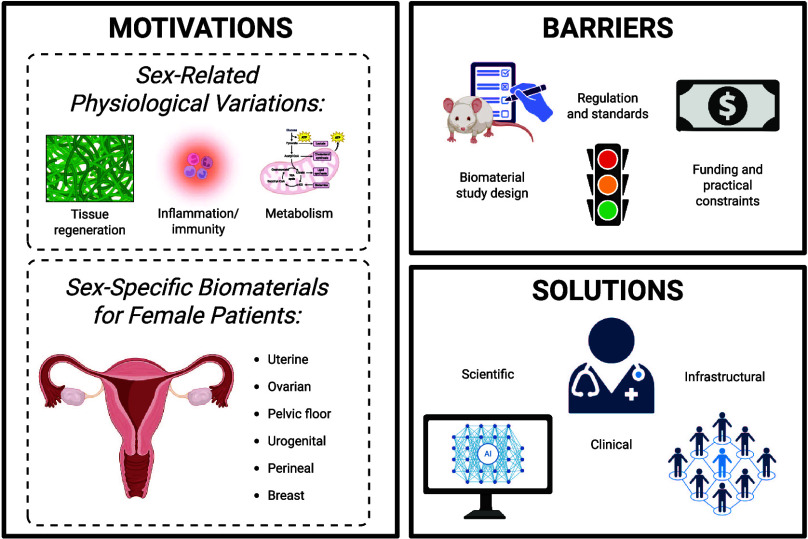

## Motivations

Biomaterials research has long focused on solutions to regenerate tissue, enable diagnoses, and deliver bioactive molecules.^1–3^ Nevertheless, the role of biological sex and sex-influenced disease status in shaping these innovations remains underexplored, representing an untapped opportunity to refine biomaterial design to meet the needs of individual patients.

### What’s “Sex” Got To Do With It?

We begin with a necessary definition of terminology. The following discussion on sex-related differences in biomaterial interactions and applications refers specifically to biological sex as a physiological factor influencing tissue composition, hormonal environment, immune responses, and relevant diseases. The focus of this article on biological sex is separate from, but does not diminish, the importance of gender identity in shaping patient-centered care.^4^ Nevertheless, the term “sex” is often inaccurately replaced with “gender” in scientific discourse, which can introduce ambiguity in describing physiological differences.^5–7^ This linguistic inaccuracy creates challenges in research and translation of biomaterials incorporating sex-related differences, limiting our ability to consistently frame and address sex-specific phenomena.

Sex-related differences in tissue structure and function are influenced by hormonal variations, genetic expression patterns, and inherent immune system differences which play pivotal roles in body–biomaterial interactions.^8^ Effects of (patho)­physiological states that vary across the lifespan further modulate these processes, thus dictating degradation kinetics, biomaterial integration, and therapeutic outcomes. However, the design of biomaterials has historically relied on generalized approaches, overlooking the variability introduced by sex.^9^ As a result, therapies fail to address the nuances of varying patient populations, limiting their clinical efficacy and scalability. While design principles for sex-specific applications can inform biomaterials development across many contexts, this perspective particularly elaborates on female-specific biomaterials given the especially underexplored potential for innovation in these areas.

### Sex-Related Physiological Variations

Sex-related differences influence a wide range of physiological processes relevant to biomaterials research, impacting tissue regeneration and inflammatory/immune responses. Understanding these sex-related differences is critical for designing biomaterials that perform optimally across patient populations.

#### Tissue Regeneration

Tissue regeneration varies significantly between sexes due to differences in hormonal regulation, cellular proliferation and other signaling pathways, and extracellular matrix (ECM) composition and turnover.^10,11^ Estrogen upregulates fibroblast proliferation, collagen synthesis, osteogenesis, and vascularization, contributing to more efficient tissue healing in premenopausal females, whereas declining estrogen levels in postmenopausal females lead to impaired regenerative capacity.^10,12–16^ Meanwhile, testosterone promotes enhanced bone mineral density and muscle (re)­generation, yet is locally metabolized into different end products for males and females—dihydrotestosterone or estrogen, respectively.^17–19^ These differences alter how biomaterials designed to facilitate tissue regeneration interact with host tissues, affecting their integration, functionality, and long-term success.

#### Inflammation/Immune Responses

Sex-related differences in inflammation and the immune system have significant implications for a body’s reaction to biomaterials and thus success of their intended application. For example, females often exhibit stronger innate and adaptive immune responses.^20,21^ Consequently, approximately 80% of autoimmune and inflammatory conditions occur in females, reflecting a markedly elevated risk relative to males.^22,23^ Therefore, females may exhibit higher rates and severity of hypersensitivity reactions with resulting failure of biomaterial implants.^24^ Emerging research suggesting sex-related differences in microbiome composition also indicates modulation of biomaterial-associated inflammation and responses to implant-associated infections.^25^

Moreover, a key sex-related genetic factor is X-chromosome inactivation (XCI), a process in which one of the two X chromosomes in females is largely silenced to balance X-linked gene dosage with males.^26^ This complex mechanism is also incompletely manifested due to a phenomenon called escape, in which at least 15% of genes on the inactivated X-chromosome persist in contributing to the expression of that trait.^27^ In particular, escape leads to differential expression of immune-regulatory genes, such as Toll-like receptor 7 (*TLR7*) and CD40 ligand (*CD40L*), which may contribute to amplified immune responses in females and thus greater susceptibility to autoimmune disease. These genetic effects interact with hormonal signaling to modulate whether and how biomaterials are perceived as foreign bodies with subsequent remodeling by the host immune system.^28,29^

In the specific case of wound healing, the process induced during any biomaterial implantation, females exhibit higher collagen deposition and faster re-epithelialization but are more prone to fibrotic scarring in response to injury or foreign materials.^30–32^ In contrast, males may experience prolonged low-grade inflammation associated with testosterone’s effects on immune signaling, which may slow healing but reduce the likelihood of excessive fibrosis.^33^ For the common application of ventral hernia repair with mesh biomaterials, female patients have been found to experience higher rates than males of adverse wound outcomes including poor healing, wound infection, and chronic postsurgical pain with associated sleep disturbances.^34,35^

#### Metabolism

Sex-related differences in metabolism can dictate the biodistribution, kinetics, and therefore efficacy of drug delivery from biomaterial-based systems.^36–39^ Importantly, females tend to exhibit a greater volume of distribution and slower clearance of liposomal nanoparticles and lipid-soluble drugs or degradation products, largely due to higher average body fat content and consequently enhanced lipid uptake.^40,41^ Conversely, water-soluble nanocarriers, degradation products, and drugs display affinity for the higher average body water content of males.

Additionally, cytochrome P450 (CYP) liver enzymes, which mediate the body’s primary drug metabolism pathways, exhibit sex-specific activity patterns.^42^ Males and females each show higher activity of distinct CYP isoforms, leading to measurable differences in drug clearance rates and overall pharmacokinetics.

These factors combine to result in sex-related disparities in metabolically related disease outcomes. For example, diabetes confers a disproportionately higher cardiovascular risk in women than in men.^43^ A large meta-analysis found that women with diabetes had a 58% higher risk of coronary heart disease mortality and a 13% higher risk of all-cause mortality compared to men with diabetes.^44^ Such differences in metabolic processing and susceptibility to disease complications further underscore the need for biomaterials designed with sex-specific pharmacokinetics and therapeutic precision in mind.

### Sex-Specific Biomaterials for Female Patients

One illustrative domain for sex-specific design with significant clinical relevance and commercial demand is biomaterials tailored for health conditions uniquely affecting patients of female sex.^45,46^ Many of these conditions, such as pelvic organ prolapse, uterine wound healing, and endometriosis, result from changes in hormones and ECM that require specialized biomaterial solutions. By addressing these sex-specific challenges, biomaterials can offer more effective, long-term therapeutic options that enhance patient quality of life.

#### Pelvic Floor Disorders

One critical area of need is pelvic floor disorders, including organ prolapse, which affects a significant proportion of postmenopausal women due to the weakening of connective tissues and muscle support.^47,48^ Traditional surgical interventions often rely on synthetic biomaterial meshes, which have been associated with complications such as erosion and chronic pain.^49^ Therefore, novel biomaterial approaches must focus on improving biocompatibility, reducing inflammatory responses, and enhancing integration with host tissues to minimize complications and ensure long-term success. Advancing these designs with bioactive materials that actively promote tissue remodeling and healing is critical to overcoming the limitations of current synthetic options.

#### Breast Reconstruction and Contraceptive Implants

Significant demand also exists for optimized biomaterials for breast modification and reconstruction following mastectomy or cosmetic procedures.^50,51^ Additionally, contraceptive implants exemplify the application of controlled release principles for drug delivery.^52^ The hormones are released for years in the case of long-acting reversible contraception like intrauterine devices, while localization of effect is crucial to minimize systemic side effects. Complementarily, another emerging area of biomaterials for family planning is ovarian tissue engineering and fertility preservation for women undergoing chemotherapy or premature ovarian failure.^53^

#### (Peri)­menopausal and Perineal Tissue Regeneration

Meanwhile, several potential biomaterials applications for female-specific conditions with widespread need remain largely underdeveloped. For example, vaginal and urethral tissue regeneration would offer improvements for the common menopausal conditions of stress urinary incontinence and vaginal atrophy.^54,55^ Declining estrogen levels during and after menopause lead to thinning of vaginal epithelium, reduced elasticity, and increased tissue fragility, often resulting in discomfort and functional impairment.^56,57^ Additionally, insufficient wound healing after severe perineal trauma during childbirth can result in chronic complications such as pain or incontinence.^58^

#### Uterine Wound Healing and Endometriosis

Beyond the pelvic floor, biomaterials also hold promise for addressing uterine wound healing following Cesarean sections and other surgeries, such as fetal intervention or fibroid removal.^59,60^ Furthermore, endometriosis, a chronic inflammatory condition in which vasculature-rich tissue of the uterine lining exists outside of the uterus, displays a recurrent nature that currently necessitates repeated surgical interventions. This application is overdue for biomaterial therapies that mitigate its recurrence and progression.^61,62^

By incorporating sex-specific considerations into biomaterial design, researchers and clinicians can develop therapies that address the distinct physiological and hormonal influences affecting patients of different sexes. Advancing these technologies not only enhances therapeutic efficacy and patient satisfaction but also promotes a more adaptive and precise approach to biomaterials-based medicine.

## Barriers

### Biomaterial Study Design

Most studies evaluate biomaterials in only a single sex, often due to concerns that biological variability—particularly hormonal fluctuations in female subjects—could confound results.^6^ While these variations do introduce additional complexity, excluding one sex from study design ultimately undermines the generalizability and applicability of biomaterials (Table 1). In reality, understanding how biomaterials perform under hormonally dynamic conditions is essential, as real-world patient populations include individuals with varying hormone levels due to age, puberty, menstrual cycles, pregnancy, or hormone therapy.^7,63–65^ Without accounting for these variables, biomaterials may be designed under conditions that do not fully represent their eventual clinical use, increasing the risk of poor clinical performance or unexpected outcomes in different sexes.

**Table 1:** Current Barriers and Proposed Solutions for Developing Biomaterials Optimized to Sex-Related Differences

Barrier	Proposed Solution	Relevant Literature
Single-sex preclinical models limiting generalizability	Standardized protocols requiring sex-balanced study designs	^212,213^
Inconsistent methodologies for biomaterial evaluation across sexes	Harmonized experimental frameworks integrating sex as a key variable	^80,214^
Lack of systematic sex-disaggregated data collection	Required reporting of sex-disaggregated data for transparency and meta-analyses	^82,215^
Regulatory frameworks lacking enforcement of testing policies for sex-related differences	Establishment of guidelines requiring sex-related evaluation in biomaterials research (e.g., Sex as a Biological Variable (SABV) and Sex and Gender Equity in Research (SAGER))	^194,216^
Funding constraints for sex-balanced studies	Targeted funding incentives and matching grants for sex-related biomaterials research	^214,217^
Limited collaboration between biomaterials scientists and clinicians	Partnerships integrating biomaterials researchers, clinicians, and industry	^80,81,112^
Sex-specific factors treated as post hoc variables	Study designs incorporating sex-related differences as predefined factors	^218,219^
Fragmented data sets hindering cross-study comparisons	Open-access data-sharing platforms following (FAIR) Findable, Accessible, Interoperable, and Reusable principles	^77,220,221^

Additionally, a lack of standardized testing protocols across research groups makes it difficult to compare results, further limiting the ability to detect sex-related variability in biomaterial performance. In other fields, significant insights have only emerged by aggregating data across multiple research groups,^66–68^ yet data sets in biomaterials research have rarely been compiled and analyzed at scale. This lack of integration, combined with inconsistent methodologies and variable outcome measures, limits the ability to systematically assess sex- and disease-specific effects.^69^ Without standardization, biomaterial properties such as degradation rates, mechanical responses, and immune interactions may appear inconsistent across studies, making it difficult to determine whether observed variations are due to true sex-related (patho)­physiological differences or artifacts of experimental design.^70^ These inconsistencies ultimately hinder the development of biomaterials optimized for sex-related differences and prevent a full understanding of how sex-specific factors influence biomaterial integration and function.

### Regulation and Standards

The U.S. National Institutes of Health (NIH) have established a policy on Sex as a Biological Variable (SABV) and guidelines on Sex and Gender Equity in Research (SAGER).^71^ However, unlike other biomedical fields for which SABV compliance is increasingly enforced, the field of biomaterials research still lacks clear expectations for complying with sex-disaggregated testing and reporting.^72,73^ Additionally, although regulatory awareness of sex differences in biomedical research has improved in recent years, this progress has not yet translated into consistent expectations for biomaterials studies.

For example, the U.S. Food and Drug Administration (FDA) released updated guidance in January 2025 entitled Evaluation of Sex Differences in Medical Device Clinical Studies.^74^ This document outlines the FDA’s expectations for the inclusion, analysis, and reporting of sex-disaggregated data in clinical research. It emphasizes the importance of enrolling a fair representation of sexes in clinical trials to detect clinically significant sex-related differences in response. The guidance also recommends collecting pharmacokinetic data on demographic differences, beginning with early phase studies to inform relevant study designs for later trials.

However, such guidelines are not currently enforced in the evaluation of biomaterials, leaving a critical gap in regulatory oversight. Without clear mandates or standardized pathways to incorporate sex-related analyses in biomaterials research, even well-defined frameworks risk a lack of implementation.

Furthermore, the lack of specific standardized testing and regulatory requirements further perpetuates gaps in understanding how biomaterials perform across different sexes. For example, many biomaterial testing protocols, such as ISO 10993 (biocompatibility evaluation), lack explicit guidance on incorporating different sexes in preclinical studies.^75^ Without enforcing a requirement for sex-disaggregated data, potential sex-specific interactions with biomaterials may remain underexplored.^76,77^

### Funding and Practical Constraints

Cost remains a major barrier to the realization of sex- and disease-specific biomaterials, as designing biomaterials for different populations often requires additional research, manufacturing customization, and testing. Limitations in available grant funding make it especially challenging to implement sex-related research, as studies designed to include sex-related comparisons require expanding the number of experimental groups, thus increasing the need for additional cells, animals, and research materials.^78,79^ These financial constraints often force researchers to prioritize single-sex studies to remain within funding constraints, further perpetuating the lack of sex-specific data in biomaterials research.

An additional challenge lies in the limited availability of sex-stratified biological samples. For example, sourcing sufficient quantities of primary cells and tissues from female donors—particularly those representing specific hormonal states such as menstruation, pregnancy, or (peri) menopause—remains logistically complex and possibly even prohibitive.^80–82^ This limitation hampers the ability to design both rigorously powered studies and biomaterials themselves that are representative of and tailored to female-specific physiology and to achieve reproducible, translatable results across the sex spectrum.

Additionally, the increased cost of scaling manufacturing processes for biomaterials tailored to sex-specific needs presents another hurdle in translating laboratory findings into clinical applications.^83^ Without targeted funding initiatives or regulatory incentives, the financial burden of biomaterials research on sex-related differences continues to slow progress toward precision biomaterial solutions.^84^

## Solutions

### Scientific

#### (Pre)­clinical Study Design

Testing biomaterials on each sex of animals or human-derived cells would provide a more comprehensive understanding of their performance across populations, ensuring balanced representation of sex-specific variables.^85^ Beyond simple inclusion, study designs should integrate and report on sex-related analyses as a predefined variable rather than a post hoc consideration.^78,86–88^ Standardizing protocols to include sex-specific considerations at all stages of biomaterial testing—cell culture, *in vivo* models, and early phase clinical trials—will ensure meaningful comparisons and applicability to different populations.

Additionally, experimental designs should account for hormonal fluctuations that could influence biomaterial interactions differently in each sex’s models.^89,90^ For example, studying biomaterial integration in female models at different phases of the menstrual cycle can provide insights into hormone-mediated regenerative or inflammatory responses. This approach will help researchers develop biomaterials that are optimized for real-world physiological variability with superior representation of potential clinical outcomes.

#### Data Generation and Sharing

To evolve beyond the fragmented approach of only assessing biomaterials within the scope of individual studies, ensuring high-quality data set integration is crucial for enabling comprehensive analyses of biomaterial performance across different sexes. Addressing this challenge requires a fundamental shift in how biomaterial data is generated, moving away from isolated, lab-specific methodologies and data storage toward standardized protocols that capture sex-specific effects.^70,91^ Achieving this goal demands rethinking study design from the outset, ensuring that data collection, structuring, and sharing are aligned for large-scale analyses. Data sets should be augmented to achieve balanced representation of each sex.^79^ Incorporating structured metadata such as documentation of these variables will further facilitate meta-analysis, reducing inconsistencies across biomaterial studies and improving comparability while avoiding overgeneralizations.^92,93^

Beyond data generation, raw data should be shared across research groups to allow for large-scale aggregation and cross-study comparisons. Standardizing data formats and ensuring adherence to FAIR (Findable, Accessible, Interoperable, and Reusable) data storage principles will facilitate data set integration and reanalysis.^94^ By compiling and harmonizing these data sets, researchers can minimize systematic biases and generate more meaningful insights into sex-related differences in biomaterial performance. Establishing open-access repositories and collaborative data-sharing networks will be key to achieving these goals, ensuring that biomaterials research can advance with greater precision.

#### AI-Driven Analysis

Integrating artificial intelligence (AI) offers an unprecedented opportunity to automate, standardize, and scale biomaterial design and evaluations, making traditional manual approaches increasingly obsolete. Machine learning (ML), a subset of AI, can enable advanced image analysis that extracts quantitative metrics from histological data sets with greater detail and efficiency than human observers, allowing reproducible comparisons of biomaterial performance across preclinical and clinical data sets.^95^ When paired with proper data aggregation practices,^96^ ML can also further uncover subtle sex-related differences in biomaterial performance that might otherwise be undetectable, facilitating predictive modeling for patient-specific therapies.^97^ These capabilities can guide the design of more effective biomaterials tailored to individual patient contexts.^98–106^ For example, ML models can predict how specific biomaterial properties interact with sex-specific hormonal profiles or disease-related inflammation.^107,108^

#### Additive Manufacturing

Once the optimized biomaterial design properties are identified, additive manufacturing methodologies like three-dimensional (3D) printing can provide precise control over biomaterial geometry, porosity, and localization of bioactive molecules.^1,109^ This capability is particularly relevant for incorporating sex-specific parameters into biomaterial design. For instance, 3D printing could enable the production of hormone-responsive scaffolds tailored for postmenopausal women, adapting to the specific vascularization and tissue integration capacities in this patient population.^110^ Similarly, materials with finely tuned microstructures could be designed to support tissue regeneration in diabetic wounds, where sex-related differences in metabolic regulation and immune response influence healing dynamics,^111^ as described in the following section.

### Clinical

#### Disease-Specific Biomaterial Solutions

Both physiologically and pathologically, sex-related factors such as hormones, genetic expression patterns, and inherent immune system differences dictate a patient’s body’s response to interactions with a biomaterial.^36,112^ These biological variables also influence the incidence, progression, manifestations, and complications of conditions such as osteoporosis, cancer, and diabetes. Such disease-driven alterations significantly impact tissue architecture, cellular dynamics, and biomaterial performance. Importantly, biomedical disciplines such as cardiology have increasingly recognized and addressed sex-related differences, yielding improved diagnostic tools and therapies tailored to specific populations.^113–116^ Embracing this perspective shift within the biomaterials field would critically enable progress toward precision medicine. Representative examples of biomaterial technologies incorporating sex-specific design features and evaluated in (pre)­clinical trials across relevant disease contexts are summarized in Table S1.

##### Osteoporosis

In the case of osteoporosis, a metabolic bone disease characterized by reduced bone density and increased fracture risk, the influence of sex hormones and therefore sex-related disparities in disease incidence are well-documented.^117^ Particularly, postmenopausal women experience a disproportionately higher rate of osteoporosis-related fractures compared to men due to declining estrogen levels. Global estimates suggest that approximately one in three women over age 50 will experience osteoporotic fractures, versus one in five men.^118,119^ Critically, hip fractures in women are associated with a resulting 20% risk of death in the year following a hip fracture, a figure that rivals or exceeds several cancers.^120–122^ Therefore, while osteoporosis is often downplayed as an inevitable experience of aging in women, in actuality it is a serious, life-threatening condition with profound consequences for morbidity and mortality. These outcomes highlight the urgent need for biomaterial designs that address the sex-specific aspects of disease progression, functionality, drug delivery efficiency, and diagnostic accuracy.^9,112,123^

The disproportionate prevalence of osteoporosis in postmenopausal women underscores significant sex-related differences in how those patients’ tissues will respond to biomaterials attempting tissue regeneration or therapeutic delivery.^124^ The interplay of hormonal decline, reduced bone density, and diminished healing capacity necessitates tailored biomaterials to improve outcomes.^125–127^ For example, biomaterials incorporating estrogen-releasing or hormone-mimetic coatings could locally modulate cell behavior and responsiveness to the biomaterial’s primary effect, counteracting estrogen-deficiency-induced impairments in bone healing and integration.

Additionally, load-responsive scaffolds tailored for osteoporotic bone could feature adaptive stiffness gradients that accommodate weaker trabecular structures, or degradation kinetics that adjust based on reduced bone remodeling rates, ensuring prolonged support and enhanced integration in postmenopausal patients.^128–130^ Given that osteoporosis is a progressive disease, biomaterials could be designed to dynamically adjust their mechanical properties over time, providing increased support as bone density declines or incorporating resorption-modulating elements that respond to ongoing remodeling deficits.

##### Cancer

Tumor microenvironments vary significantly between sexes due to previously discussed factors such as sex-related differences in vascularization, immune responses, and drug metabolism.^131^ Given these differences, biomaterials designed for cancer therapy could incorporate immune-modulating coatings that adjust inflammatory responses based on the hormonal environment of the tumor.^132,133^ Such materials could optimize tumor-targeting efficacy or biomaterial integration into cancer-altered tissues by harnessing immune activation or promoting immune tolerance when necessary for the intended mechanism of action.

Additionally, biomaterials for drug delivery in hormone-responsive cancers could leverage sex-specific hormonal biomarkers to enhance targeting precision.^134^ For instance, nanoparticle-based drug carriers could be engineered to respond to estrogen or testosterone levels, releasing therapeutic agents in a controlled manner based on the tumor’s microenvironment,^40^ thus enabling localized and controlled drug release in response to tumor-associated hormone fluctuations. This function would be critical in estrogen- or progesterone-receptor positive breast cancer as well as androgen-driven prostate tumors.^135,136^ Such an approach could improve drug retention at the tumor site and minimize off-target effects, addressing sex-related differences in drug metabolism and immune interactions with biomaterials.^73,137^ Alternatively, for the forms of lung and pancreatic cancer exhibiting sex-related differences in drug metabolism and immune response,^138–140^ biomaterials exhibiting adaptive release rates could optimize therapy differentially in male or female patients.

##### Autoimmune/Inflammatory Conditions

Conditions like rheumatoid arthritis exhibit sex-related differences in prevalence and therapeutic efficacy, altering the performance of biomaterials.^141–144^ Therefore, biomaterials meant to regenerate tissue or deliver biomolecules in female patients with one or multiple autoimmune/inflammatory conditions could incorporate immunomodulatory coatings that selectively dampen excessive immune activation locally, mitigating excessive fibrosis while preserving regenerative signaling and drug delivery capacity, thus promoting improved healing in high-inflammation environments.^145–148^ Given that these diseases are often progressive,^149^ smart biomaterials could be engineered to release anti-inflammatory compounds over time or in response to immune system fluctuations, ensuring sustained efficacy and reducing the long-term burden of chronic inflammation on the biomaterial’s targeted functionality. Conversely, in men, for whom chronic low-grade inflammation is more prevalent, biomaterials could be designed to gradually enhance immune stimulation or promote macrophage polarization toward a reparative phenotype, improving long-term biomaterial integration and function.

##### Diabetes

The pathophysiology of diabetes differs between sexes due to hormonal, metabolic, and immune factors.^150–152^ For example, the differential distribution of fat—subcutaneously in females, viscerally in males—influences inflammation, drug metabolism, and tissue repair.^153^ These factors in turn compound the characteristic complications of wound healing in diabetes resulting from chronic hyperglycemia.^154^ Given these differences, biomaterials designed for diabetic patients of different sexes could incorporate anti-inflammatory or immunomodulatory coatings that could account for the baseline variations in inflammatory/immune phenomena.^155^

For drug delivery applications of biomaterials for diabetes, biomaterial designs must account for the sex-related variations in vascularization, which may determine bioavailability of the biomolecule to the target organ.^156^ Different dosages of pro-angiogenic factors may also need to be included in biomaterials for female patients to accommodate their microcirculation, given their higher propensity for microvascular complications,^157,158^ while incorporating local vasodilatory agents into biomaterial implants for male patients to counteract their higher risk of cardiovascular complications.^159^

##### Female-Specific Conditions

Sex-specific biomaterial design principles offer transformative potential in addressing a range of conditions, including those characteristic of patients with female reproductive anatomy.^160,161^

##### Pelvic Floor and Urogenital Repair

For pelvic floor disorders, next-generation biomaterial scaffolds with enhanced bioactivity and mechanical adaptability could promote tissue integration, reduce foreign body reactions, and restore structural integrity. Incorporating localized growth factors or estrogen-mimetic compounds may further enhance tissue regeneration and reduce recurrence rates.^162^

In the realm of vaginal and urethral repair, current clinical solutions remain limited, highlighting an opportunity for biomaterial innovations.^54,163^ Hormone-responsive hydrogels, injectable biomaterials, or localized implants could support epithelial regeneration, enhance collagen production, and maintain tissue hydration. Similarly, biodegradable bulking agents for urethral support could improve continence by reinforcing tissue structure while minimizing complications associated with synthetic materials.

##### Uterine and Perineal Wound Healing

Additionally, fibrosis occurs after incision in the uterine muscle during surgeries such as Cesarean (C−) sections, fetal surgery, and fibroid removal.^164^ The resulting scar can lead to chronic pelvic pain and serious complications in subsequent pregnancies. Instead, biodegradable biomaterial scaffolds could play a key role in uterine wound healing by preventing adhesions and promoting regeneration. Injectable hydrogels and electrospun nanofibrous scaffolds could also aid in perineal wound healing, particularly in cases of severe tearing or fistula formation.^165^

##### Endometriosis and Localized Drug Delivery

Furthermore, for chronic gynecological conditions with limited current therapeutic options, biomaterials offer promising avenues for improving treatment. Endometriosis, a highly recurrent inflammatory disease, could be managed using implantable biomaterials that release localized therapies in response to menstrual cycle-regulating hormones or heme detection, reducing lesion regrowth postsurgery.^61^

##### Contraception and Fertility

In the application of family planning, smart biomaterials could similarly enable responsive drug delivery for contraception, ensuring precise hormone release aligned with endogenous fluctuations.^166–169^ Meanwhile, for fertility preservation and infertility treatments, biomaterial scaffolds mimicking ovarian stromal tissue could support *in vitro* follicle maturation, providing a platform for supporting follicle survival in both research (on-chip) and clinical settings.^170^

##### Breast Reconstruction

Finally, biomaterials for breast tissue engineering could enhance reconstruction outcomes following mastectomy or cosmetic procedures.^50^ Biodegradable scaffolds supporting adipose and glandular tissue regeneration could minimize the need for permanent implants, improving long-term functional and aesthetic results. These innovations hold the potential to significantly improve postsurgical recovery and patient quality of life.

##### Additional Examples

In the biomaterials application of cardiac stents, which are inserted in blood vessels to restore blood flow upon blockage, a large-scale clinical trial recently determined the sex-related differences in key device performance outcomes.^171^ Females experienced a higher rate of myocardial infarction (colloquially known as a “heart attack”), which cardiac stents intend to prevent and treat. Meanwhile, males received more repetitions of the procedures to reopen the blocked blood vessel. Given the larger anatomical diameter of male blood vessels,^172^ consequent distinctions in the biomechanical environment, and previously discussed inflammatory tendencies, sex-specific coatings could be designed for the stent surface to more optimally mitigate clotting and promote native endothelial cell migration for female and male patients, respectively.

Furthermore, for biomaterials that interface with nerve tissue to provide temporary or permanent measurements or therapeutic stimulation, male patients have a higher density of neuroimmune microglia cells and therefore more neuroinflammatory reactivity that can cause dysfunctional device encapsulation and disrupt signal transmission.^173^ Neural interface biomaterials for males could be tailored with immunomodulatory, antifibrotic surface chemistries that dampen such aggressive encapsulation.^174^ Meanwhile, biomechanically matched interface surfaces that support natural integration without oversuppressing beneficial immune responses would benefit female patients.^175–177^ This sex-specific approach would ensure optimized biocompatibility and functional longevity across sexes.

### Infrastructural

#### Collaboration

Collaborating with physician-scientists who witness firsthand the limitations of biomaterials in addressing sex-related differences is essential to ensuring clinical relevance.^46,178,179^ Engaging clinicians in a continuous feedback loop throughout the biomaterial design process—before, during, and after market introduction—can refine innovations based on real-world, sex-related variability, bridging the gap between research and patient outcomes.^180^

Beyond academic partnerships, industry engagement is critical to accelerating biomaterials research that incorporates sex-related considerations.^181^ The biomaterials market has yet to fully capitalize on the demand for sex-specific products, particularly in areas such as osteoporosis treatments, cardiovascular implants, and drug delivery systems for hormone-sensitive conditions. Encouraging investment in biomaterials tailored to different physiological profiles can assist with closing this gap, making sex-specific biomaterials research not only a scientific necessity but also an as-yet largely untapped commercial opportunity that enhances long-term healthcare outcomes.

Additionally, concrete implementation pathways are critical to translating interdisciplinary collaboration into lasting change. For example, *The Lancet Women and Cardiovascular Disease Commission* has served as a high-impact model by aligning academic, clinical, and policy stakeholders to generate sex- and gender-specific research priorities, establish reporting standards, and advocate for institutional reforms.^182^ Similar consortia could be leveraged in biomaterials to set research agendas, create shared repositories of sex-disaggregated data, and inform guidelines for sex-specific biomaterial development.

Existing initiatives such as the NIH *Office of Research on Women’s Health* (ORWH) and the European Commission’s *Horizon Europe* framework program also provide structural models and funding mechanisms that could be adapted or expanded to support sex-specific biomaterials research. For example, ORWH’s *Strategic Plan for Women’s Health Research* and *Building Interdisciplinary Research Careers in Women’s Health* (BIRCWH) programs explicitly support translational and collaborative projects across scientific disciplines.^183–190^ Likewise, Horizon-funded consortia such as *GENDER-NET Plus* have established templates for incorporating sex and gender analysis into large-scale biomedical projects.^191^ Establishing such structured frameworks would ensure that collaborations extend beyond dialogue and lead to systemic improvements in research and clinical application.

#### Funding and Regulation

Addressing the structural challenges that hinder the integration of sex-related differences in biomaterials research requires targeted changes in funding priorities and regulatory frameworks.^192^ Fortunately, a template for these measures already exists in the form of the SAGER guidelines.^193–195^ Ensuring financial and institutional support for studies investigating sex-related differences will improve the reproducibility, clinical applicability, and long-term impact of sex-specific biomaterial innovations.

To overcome the current barriers, innovative funding models are necessary to support the increased costs associated with incorporating sex-related differences in research. Since biomaterial studies that assess different sexes require additional experimental groups, more extensive analyses, and greater statistical power, research budgets must reflect these demands.^196^ Funding agencies should establish dedicated grants or matching funds to support studies that explicitly investigate sex as a variable in biomaterials development. Specialized initiatives can be developed to reevaluate key studies that were originally conducted in only one sex, expanding their scope to include comparative analyses of sex-related differences. These efforts would not only improve the reproducibility of findings but also ensure that developing therapeutic strategies are applicable across populations by addressing sex-related variability in biomaterials research.^197^

While tailored sex-specific biomaterials development would incur higher upfront costs due to additional preclinical testing and stratified clinical studies, these investments can be offset and eventually surpassed by longer-term benefits.^198–200^ Devices and therapies tailored to sex-specific physiological differences are more likely to achieve sustained efficacy, reduce rates of failure and revision procedures, and minimize chronic adverse effects, thereby lowering downstream healthcare expenditures.^200–205^ For example, precision strategies in oncology have demonstrated that stratified approaches often lead to improved clinical outcomes and more efficient use of healthcare resources over time.^206^

Creative public-private partnership models and financial healthcare reforms have been proposed with the goal of facilitating the development, implementation, and adoption of novel technologies.^206–208^ These approaches support shared investment in innovation, equitable risk distribution, and long-term value realization. Analogous frameworks could support sex-specific biomaterials development by encouraging the pooling of financial, infrastructural, and data resources across research institutions, public health agencies, and private industry. Specifically, harmonized use of preclinical testing platforms and patient data registries with shared control groups would reduce duplicated infrastructure and experimentation.^209,210^ Such strategies may also help align economic incentives across payers, regulators, and developers to ensure that the added value of sex-specific approaches is recognized and reimbursed accordingly.^211^

Additionally, regulatory frameworks should evolve to enforce requirements for balanced preclinical and clinical representation among the sexes, accompanied by clear guidelines for data disaggregation and reporting to ensure transparency and reproducibility,^72,76,200^ thus reducing the risks of biased or incomplete data informing product development and regulatory approval.

## Outlook

The incorporation of sex-related differences into biomaterials research is a critical step toward advancing precision medicine and ensuring that biomaterials perform optimally across diverse patient populations. As this perspective has outlined, sex influences key biological processes—from tissue regeneration and immune response to drug metabolism—yet biomaterials research has historically overlooked these factors. A shift toward sex-specific biomaterial design will require fundamental changes in study design, data integration, regulatory standards, and interdisciplinary collaboration.

To achieve this, biomaterials research must move beyond the traditional “one-size-fits-all” paradigm and instead embrace data-driven, sex-specific strategies that improve therapeutic efficacy and patient outcomes. Standardizing preclinical testing frameworks to include sex-specific analyses, developing biomaterials that account for sex-specific physiological differences, and leveraging AI and computational tools to analyze biomaterial–host interactions will be essential steps in this transition. Additionally, regulatory agencies and funding bodies should specifically incentivize biomaterials research practices for investigating sex-related differences, ensuring that future innovations are designed to be both scientifically rigorous and truly patient-centered.

## Supplementary Material



## Notes

Examples of Sex-Specific Biomaterials in Clinical Trials (Table S1) (PDF)
