# Supplementary material for: Toward Sex-Specific Biomaterials Innovation: A Perspective
Source: ACS Biomater Sci Eng. 2025 Aug 20;11(9):5131–44. doi: 10.1021/acsbiomaterials.5c00342 (PMC12421524; doi:10.1021/acsbiomaterials.5c00342)
Supplement: Supplementary file 1 [file ab5c00342_si_001.pdf]

Supporting Information for Publication

Toward Sex-Specific Biomaterials Innovation: A Perspective

Gerry L. Koons, MD, PhD[1,2,*]

[1]Regenerative Biomaterials Research Group, Research Institute for Medical Innovation, Radboud University Medical Center, Nijmegen, 6525 GA, The Netherlands

[2]Computational Pathology Research Group, Research Institute for Medical Innovation, Radboud University Medical Center, Nijmegen, 6525 GA, The Netherlands

*Corresponding author: Gerry.Koons@radboudumc.nl

Table S1. Examples of Sex-Specific Biomaterials in Clinical Trials. Biomaterials that are approved and in clinical use (green), undergoing (pre) clinical trials (yellow), or did not reach/withdrawn from regulatory approval/sale (red). CЄ mark = Conformité Européenne (European Economic Area regulatory approval); FDA = U.S. Food and Drug Administration; N/A = not applicable; RCT = randomized controlled trial.

Clinical Application	Biomaterial (Product)	Sex-Specific Design Features	Clinical Trial/Regulatory Status	Key Outcomes	Translational Challenges	Relevant Literature
Osteoporosis	Mesoporous silica nanoparticles with estradiol release on titanium	Female-predominant disease burden	Preclinical (*in vitro*) models	Potential improvement in postmenopausal osteogenesis/osteointegration	Human trials needed, regulatory complexity (drug–device combination), clinical adoption	1
Cancer (Breast)	Polylactic acid/polyglycolic acid with titanium clips (implantable radiographic marker)		Recall/ban in 2024 following postmarket complications	High complication rate (erosion, pain, migration, infection, rash) with subsequent need for removal; polymer absorption within 2 years	Regulatory recall and litigation	2, 3
	Silicone with anti-estrogen release (tube)		Preclinical (animal) models	Potential improvement in delaying tumor recurrence	Controlled release requirements	4
	Poly(DL-lactic-co-glycolic acid) (PLGA) nanoparticles with chemotherapeutic release		Preclinical (animal) models	Potential improvement in targeted tumor uptake and reduced systemic toxicity	Regulatory complexity (drug–device combination), scalability, patient selectivity	5, 6
Cancer (Prostate)	Polyethylene glycol (injectable hydrogel spacer)	Male-specific anatomy	Completed RCTs, holds FDA clearance and CE mark	High improvement in reducing rectum radiation dose; higher sexual function preservation, gradual resorption without safety issues	Complex delivery, high cost	7-12
Autoimmune/Inflammatory	*[No reported sex-specific biomaterials/products]*					-
Diabetes	*[No reported sex-specific biomaterials/products]*					-
Pelvic Floor Repair	Polypropylene (transvaginal mesh)	Female-specific anatomy	Recall/ban in 2019 following postmarket complications	High complication rate (erosion, pain); lower prolapse recurrence	Regulatory bans and litigation	13-15
	Porcine dermis (biologic grafts/absorbable mesh)		Evaluated in RCTs, did not earn regulatory approval	No improvement in efficacy vs. native tissue repair; higher prolapse recurrence, lower immune reaction	Non-superior efficacy, limited durability and high cost	16, 17
Urogenital Repair	Polypropylene (mid-urethral sling)		Completed RCTs, holds FDA clearance and CE mark	High improvement in continence; low rate of mesh exposures	Requires surgical expertise	18-21
	Polyacrylamide (hydrogel bulking agent)		Completed RCTs, holds FDA clearance and CE mark	High improvement in continence; low rate of complications	Required injection technique, may later require re-injection	22-25
	Small intestinal submucosa matrix (graft)		Phase I/II	Potential improvement in epithelial/muscle integration; long duration of structural maintenance and normal sexual function	Regulatory complexity (cell therapy–device combination), scalability	26
	Autologous muscle-derived stem cells on collagen (injectable scaffold)		Phase I/II	Modest improvement in continence; no major reported safety issues	Variable efficacy, cell harvest/processing complexity and regulatory classification	27, 28
Perineal Wound Healing	Platelet-rich plasma with growth factors (topical biologic gel)		Phase II	Potential improvement in wound pain and healing speed	Inconsistent efficacy, standardization of material preparation	29-32
	Collagen hydrolysate (injection)		Pilot RCT	Potential improvement in chronic wound pain and appearance	Unclear mechanism, infection/allergen risk	33
	Cyanoacrylate (skin adhesive)		Ongoing RCT (phase N/A)	Potential improvement in pain and procedural efficiency	Mechanical stress (tension/friction) of anatomical site	34-42
Vaginal Tissue Engineering	Autologous vaginal cells on porcine intestinal mucosa (implantable scaffold)		Pilot cohort with 4 patients	Functional vaginal organ (normal structure and sexual function); no long-term complications	Rare application, specialized preparation facilities and high cost	43
Uterine Adhesions (Wound Healing and Endometriosis)	Oxidized regenerated cellulose (absorbable mesh barrier)		Completed RCTs, holds FDA clearance and CE mark	High improvement in intrauterine adhesion incidence and complications; high subsequent fertility rates	Requires thorough hemostasis for placement, rapid dissolution before epithelialization	44
	Hyaluronic acid (auto-crosslinked gel)		Phase III	Moderate improvement in intrauterine adhesion incidence; high subsequent fertility rates	Requires thorough hemostasis for placement, limited regulatory clearance/guidance	45, 46
	Human umbilical cord stem cells on collagen		Phase I/II	Moderate improvement in endometrial thickness; high subsequent fertility rates, no major reported safety issues	Regulatory complexity (cell therapy–device combination), cycle-dependent insertion timing, high cost	47-50
	Decellularized placental membrane (intrauterine amnion graft)		Pilot case series	Implantation feasibility; potential endometrial regeneration and adhesion reduction	Fragile handling, disease screening	51-55
	Silicone ± with estrogen release (sheet or plate or stent)		Pilot case series	Potential improvement in intrauterine adhesion incidence; no major reported safety issues	Site retention, insertion technique	56-58
	Polytetrafluoroethylene (nonabsorbable membrane)		Limited studies, not widely approved/clinically adopted	Potential improvement in intrauterine adhesion incidence	Requires subsequent removal surgery	59-60
Drug Delivery (Endometriosis)	Poly(DL-lactic-co-glycolic acid) (PLGA) nanoparticles with aromatase-inhibitor release		Preclinical (animal) models	Potential improvement in endometriosis lesion size	Human trials needed, regulatory complexity (drug–device combination), clinical adoption	61-62
Contraception (Female)	Polyethylene with progestin release (hormonal intrauterine device [IUD])		Completed RCTs, holds FDA clearance and CE mark	>99% effectiveness in preventing pregnancy for 5+ years and improvement in heavy menstrual bleeding; rare risk of uterine perforation	Selective cancer risk modulation (increased for breast, decreased for gynecologic)	63-67
	Polyethylene glycol with progestin release (subdermal rod)		Completed RCTs, holds FDA clearance and CE mark	>99% effectiveness in preventing pregnancy for 3+ years; common risk of irregular bleeding/spotting, rare risk of implant insertion/migration/ removal complications	Systemic hormonal side effects	68-73
	Ethylene vinyl acetate with combined estrogen/progestin release (vaginal ring)		Completed RCTs, holds FDA clearance and CE mark	>91% effectiveness with typical use in preventing pregnancy for 1 month; moderate risk of stroke/thromboembolism	Storage requirements (temperature sensitivity), user dependency	74-77
	Copper (non-hormonal IUD)		Completed RCTs, holds FDA clearance and CE mark	>99% effectiveness in preventing pregnancy for 10+ years; heavier menstrual bleeding and rare risk of uterine perforation	Increased menstrual bleeding, pain upon device insertion	65, 78-81
	Stainless steel, polyethylene terephthalate, nickel-titanium alloy (coil implant)		Voluntary sale discontinuation following postmarket complications	>99% effectiveness in preventing pregnancy permanently; high complication rate (pain, bleeding, autoimmune-like symptoms) with subsequent need for removal	Regulatory restriction and litigation; complexity of removal (no standardized technique, potential fragmentation), imaging limitations	82
Contraception (Male)	Styrene–maleic anhydride copolymer (injectable gel)	Male-specific anatomy	Phase III	>97% effectiveness in preventing pregnancy for 10 years; no major reported safety issues, potential for reversibility	Regulatory delays, socio-cultural acceptance	83-86
Fertility (Female)	Hyaluron-enriched embryo transfer medium ("glue" during in vitro fertilization)	Female-specific anatomy	Completed RCTs, holds FDA clearance and CE mark	High improvement in implantation rates and subsequent live births; no major reported safety issues beyond increase in multiple gestations	Variable composition, high cost	87-95
	Decellularized ovarian tissue (artificial ovary)		Preclinical (animal) models	Potential improvement in follicle survival and maturation	Regulatory complexity (advanced tissue therapy), disease screening, cycle-dependent function	96-99
	Ovarian cells in alginate		Preclinical (animal) models	Potential survival and function of encapsulated cells	Regulatory complexity (cell therapy–device combination), biological complexity (hormonal feedback, cell exhaustion with need for replacement)	100-102
Fertility (Male)	Sperm cells in alginate	Male-specific physiology	Preclinical (*in vitro*) models	Unclear improvement in sperm viability and DNA preservation	Low demand (high efficacy of current standard)	103, 104
Breast Reconstruction	*[Described in other literature]*					105-108

References

(1) Hu, Y.; Cai, K.; Luo, Z.; Jandt, K. D. Layer-by-Layer Assembly of β-Estradiol Loaded Mesoporous Silica Nanoparticles on Titanium Substrates and Its Implication for Bone Homeostasis. *Adv. Mater. Deerfield Beach Fla* **2010**, *22* (37), 4146–4150. https://doi.org/10.1002/adma.201000854.

(2) Kaufman, C. S.; Cross, M. J.; Barone, J. L.; Dekhne, N. S.; Devisetty, K.; Dilworth, J. T.; Edmonson, D. A.; Eladoumikdachi, F. G.; Gass, J. S.; Hall, W. H.; Hong, R. L.; Kuske, R. R.; Patton, B. J.; Perelson, C.; Phillips, R. F.; Smith, A. B.; Smith, L. A.; Tafra, L.; Lebovic, G. S. A Three-Dimensional Bioabsorbable Tissue Marker for Volume Replacement and Radiation Planning: A Multicenter Study of Surgical and Patient-Reported Outcomes for 818 Patients with Breast Cancer. *Ann. Surg. Oncol.* **2021**, *28* (5), 2529–2542. https://doi.org/10.1245/s10434-020-09271-2.

(3) Han, H. R.; Manasyan, A.; Kang, I.; Stanton, E.; Beriwal, S.; Daar, D. A.; Ling, D. C. Can We Have the Best of Both Worlds? Considerations for Combining Oncoplastic Reconstruction With Partial Breast Irradiation. *Pract. Radiat. Oncol.* **2025**. https://doi.org/10.1016/j.prro.2025.02.008.

(4) Park, J.; Thomas, S.; Zhong, A. Y.; Wolfe, A. R.; Krings, G.; Terranova-Barberio, M.; Pawlowska, N.; Benet, L. Z.; Munster, P. N. Local Delivery of Hormonal Therapy with Silastic Tubing for Prevention and Treatment of Breast Cancer. *Sci. Rep.* **2018**, *8* (1), 92. https://doi.org/10.1038/s41598-017-18436-1.

(5) Pandey, S. K.; Patel, D. K.; Maurya, A. K.; Thakur, R.; Mishra, D. P.; Vinayak, M.; Haldar, C.; Maiti, P. Controlled Release of Drug and Better Bioavailability Using Poly(Lactic Acid-Co-Glycolic Acid) Nanoparticles. *Int. J. Biol. Macromol.* **2016**, *89*, 99–110. https://doi.org/10.1016/j.ijbiomac.2016.04.065.

(6) Di Gregorio, E.; Romiti, C.; Di Lorenzo, A.; Cavallo, F.; Ferrauto, G.; Conti, L. RGD_PLGA Nanoparticles with Docetaxel: A Route for Improving Drug Efficiency and Reducing Toxicity in Breast Cancer Treatment. *Cancers* **2023**, *15* (1). https://doi.org/10.3390/cancers15010008.

(7) Mariados, N.; Sylvester, J.; Shah, D.; Karsh, L.; Hudes, R.; Beyer, D.; Kurtzman, S.; Bogart, J.; Hsi, R. A.; Kos, M.; Ellis, R.; Logsdon, M.; Zimberg, S.; Forsythe, K.; Zhang, H.; Soffen, E.; Francke, P.; Mantz, C.; Rossi, P.; DeWeese, T.; Hamstra, D. A.; Bosch, W.; Gay, H.; Michalski, J. Hydrogel Spacer Prospective Multicenter Randomized Controlled Pivotal Trial: Dosimetric and Clinical Effects of Perirectal Spacer Application in Men Undergoing Prostate Image Guided Intensity Modulated Radiation Therapy. *Int. J. Radiat. Oncol.* **2015**, *92* (5), 971–977. https://doi.org/10.1016/j.ijrobp.2015.04.030.

(8) Karsh, L. I.; Gross, E. T.; Pieczonka, C. M.; Aliotta, P. J.; Skomra, C. J.; Ponsky, L. E.; Nieh, P. T.; Han, M.; Hamstra, D. A.; Shore, N. D. Absorbable Hydrogel Spacer Use in Prostate Radiotherapy: A Comprehensive Review of Phase 3 Clinical Trial Published Data. *Urology* **2018**, *115*, 39–44. https://doi.org/10.1016/j.urology.2017.11.016.

(9) Folkert, M. R.; Shin, Y. E.; Rojanasarot, S.; Hathout, L.; Ezekekwu, E.; Vannan, D. Real-World Medical Device Reports of SpaceOAR Hydrogel Spacer: Analysis of The FDA Manufacturer and User Facility Device Experience (MAUDE) Database. *Adv. Radiat. Oncol.* **2025**, 101824. https://doi.org/10.1016/j.adro.2025.101824.

(10) Miller, L. E.; Efstathiou, J. A.; Bhattacharyya, S. K.; Payne, H. A.; Woodward, E.; Pinkawa, M. Association of the Placement of a Perirectal Hydrogel Spacer With the Clinical Outcomes of Men Receiving Radiotherapy for Prostate Cancer: A Systematic Review and Meta-Analysis. *JAMA Netw. Open* **2020**, *3* (6), e208221–e208221. https://doi.org/10.1001/jamanetworkopen.2020.8221.

(11) Navaratnam, A.; Cumsky, J.; Abdul-Muhsin, H.; Gagneur, J.; Shen, J.; Kosiorek, H.; Golafshar, M.; Kawashima, A.; Wong, W.; Ferrigni, R.; Humphreys, M. R. Assessment of Polyethylene Glycol Hydrogel Spacer and Its Effect on Rectal Radiation Dose in Prostate Cancer Patients Receiving Proton Beam Radiation Therapy. *Adv. Radiat. Oncol.* **2020**, *5* (1), 92–100. https://doi.org/10.1016/j.adro.2019.08.007.

(12) Folkert, M. R.; Sato, R.; Yu, J. B.; Vannan, D.; Bhattacharyya, S.; Noriega, C.; Hamstra, D. A. Bowel Disorder Incidence and Rectal Spacer Use in Patients With Prostate Cancer Undergoing Radiotherapy. *JAMA Netw. Open* **2025**, *8* (3), e250491–e250491. https://doi.org/10.1001/jamanetworkopen.2025.0491.

(13) Maher, C. F.; Yeung, E.; Chen, Z.; Masel, A.; Smith, D.; Lourie, R.; Devan, S. M.; Rasch, R.; Uwins, P.; Blakey, I.; Boyle, G. M.; Jones, L. Pathogenesis of Polypropylene Mesh Complications in Female Pelvic Floor Surgery. *Am. J. Obstet. Gynecol.* **2025**. https://doi.org/10.1016/j.ajog.2025.03.009.

(14) Seifalian, A.; Basma, Z.; Digesu, A.; Khullar, V. Polypropylene Pelvic Mesh: What Went Wrong and What Will Be of the Future? *Biomedicines* **2023**, *11* (3). https://doi.org/10.3390/biomedicines11030741.

(15) Mangir, N.; Aldemir Dikici, B.; Chapple, C. R.; MacNeil, S. Landmarks in Vaginal Mesh Development: Polypropylene Mesh for Treatment of SUI and POP. *Nat. Rev. Urol.* **2019**, *16* (11), 675–689. https://doi.org/10.1038/s41585-019-0230-2.

(16) Gigliobianco, G.; Roman Regueros, S.; Osman, N. I.; Bissoli, J.; Bullock, A. J.; Chapple, C. R.; MacNeil, S. Biomaterials for Pelvic Floor Reconstructive Surgery: How Can We Do Better? *BioMed Res. Int.* **2015**, *2015* (1), 968087. https://doi.org/10.1155/2015/968087.

(17) Shiroud Heidari, B.; Dodda, J. M.; El-Khordagui, L. K.; Focarete, M. L.; Maroti, P.; Toth, L.; Pacilio, S.; El-Habashy, S. E.; Boateng, J.; Catanzano, O.; Sahai, N.; Mou, L.; Zheng, M. Emerging Materials and Technologies for Advancing Bioresorbable Surgical Meshes. *Acta Biomater.* **2024**, *184*, 1–21. https://doi.org/10.1016/j.actbio.2024.06.012.

(18) Chmaj-Wierzchowska, K.; Raba, G.; Dykczyński, P.; Wilczak, M.; Turlakiewicz, K.; Latańska, I.; Sujka, W. Clinical Outcomes of Mid-Urethral Sling (MUS) Procedures for the Treatment of Female Urinary Incontinence: A Multicenter Study. *J. Clin. Med.* **2022**, *11* (22). https://doi.org/10.3390/jcm11226656.

(19) Garcia, C.; Homewood, D.; Gani, J.; O’Connell, H. E. Perspectives on Technology: Urethral Slings in a Post-Mesh World. *BJU Int.* **2024**, *134* (3), 337–350. https://doi.org/10.1111/bju.16388.

(20) MacCraith, E.; O’Brien, F. J.; Davis, N. F. Biodegradable Materials for Surgical Management of Stress Urinary Incontinence: A Narrative Review. *Eur. J. Obstet. Gynecol. Reprod. Biol.* **2021**, *259*, 153–160. https://doi.org/10.1016/j.ejogrb.2021.02.024.

(21) Heesakkers, J. P. F. A.; Roovers, J.-P.; Schraffordt Koops, S. E. Surgical Treatment of Female Stress Incontinence: Impact of Changed Views on Polypropylene. *Tijdschr. Voor Urol.* **2021**, *11* (6), 121–129. https://doi.org/10.1007/s13629-021-00338-7.

(22) Tam, J.; Koenig, H.; Popat, S.; Sparks, D.; Lucioni, A.; Kobashi, K.; Lee, U. PD06-01 REAL WORLD OUTCOMES OF URETHRAL INJECTION OF POLYACRYLAMIDE HYDROGEL (BULKAMID®) FOR STRESS URINARY INCONTINENCE WITH INTRINSIC SPHINCTER DEFICIENCY, PRIOR URETHRAL SURGERY, OR PRIOR MESH SLING EXCISION. *J. Urol.* **2022**, *207* (Supplement 5), e92.

(23) Itkonen Freitas, A.-M.; Isaksson, C.; Rahkola-Soisalo, P.; Tulokas, S.; Mentula, M.; Mikkola, T. S. Tension-Free Vaginal Tape and Polyacrylamide Hydrogel Injection for Primary Stress Urinary Incontinence: 3-Year Followup from a Randomized Clinical Trial. *J. Urol.* **2022**, *208* (3), 658–667.

(24) Lemmon, B.; Cardozo, L.; Bray, R.; Cortes, E. Retrospective Analysis of the Efficacy and Safety of Polyacrylamide Hydrogel (Bulkamid®) Peri-Urethral Bulking Injection at the Time of Pelvic Floor Repair in Women with Pelvic Organ Prolapse and Urodynamic Stress

Incontinence.A Pilot Study. *Continence* **2024**, *10*, 101221. https://doi.org/10.1016/j.cont.2024.101221.
(25) Eddib, H.; Lee, J.; Olafuyi, O.; Eddib, A. The Efficacy of Polyacrylamide Hydrogel (Bulkamid) Transurethral Injection System: The Outcome of Short-Term Follow-up of 75 Cases. *Am. J. Obstet. Gynecol.* **2023**, *228* (3), S896. https://doi.org/10.1016/j.ajog.2022.12.159.
(26) Huang, L.-P.; Liu, Y.; Li, Q.-J.; Zhang, W.-Q.; Wu, C.-Y.; Zhao, L.-M.; Xie, H.-Q. A Modified Small Intestinal Submucosa Patch with Multifunction to Promote Scarless Repair and Reinvigoration of Urethra. *Adv. Healthc. Mater.* **2023**, *12* (23), 2300519. https://doi.org/10.1002/adhm.202300519.
(27) Jankowski, R. J.; Tu, L. M.; Carlson, C.; Robert, M.; Carlson, K.; Quinlan, D.; Eisenhardt, A.; Chen, M.; Snyder, S.; Pruchnic, R.; Chancellor, M.; Dmochowski, R.; Kaufman, M. R.; Carr, L. A Double-Blind, Randomized, Placebo-Controlled Clinical Trial Evaluating the Safety and Efficacy of Autologous Muscle Derived Cells in Female Subjects with Stress Urinary Incontinence. *Int. Urol. Nephrol.* **2018**, *50* (12), 2153–2165. https://doi.org/10.1007/s11255-018-2005-8.
(28) Kaufman, M. R. Contemporary Application of Autologous Muscle-Derived Cells for Urinary Sphincter Regeneration. *World J. Urol.* **2020**, *38* (9), 2095–2099. https://doi.org/10.1007/s00345-019-03018-9.
(29) Akhoundova, F.; Schumacher, F.; Léger, M.; Berndt, S.; Martinez de Tejada, B.; Abdulcadir, J. Use of Autologous Platelet Rich Plasma (A-PRP) for Postpartum Perineal Repair Failure: A Case Report. *J. Pers. Med.* **2022**, *12* (11). https://doi.org/10.3390/jpm12111917.
(30) Streit-Ciećkiewicz, D.; Kołodyńska, A.; Futyma-Gąbka, K.; Grzybowska, M. E.; Gołacki, J.; Futyma, K. Platelet Rich Plasma in Gynecology—Discovering Undiscovered—Review. *Int. J. Environ. Res. Public. Health* **2022**, *19* (9). https://doi.org/10.3390/ijerph19095284.
(31) Omar, S. S.; Elmulla, K. F.; AboKhadr, N. A.; Badawy, A. A.; Ramadan, E. N.; Hassouna, A. M.; Heikal, L. A.; Arafat, W. O. Comparable Efficacy of Submucosal Platelet-Rich Plasma and Combined Platelet-Rich Plasma Noncrosslinked Hyaluronic Acid Injections in Vulvovaginal Atrophy: A Cancer Survivorship Issue. *J. Womens Health* **2023**, *32* (9), 1006–1020. https://doi.org/10.1089/jwh.2023.0144.
(32) Dwi, K. D.; Mardiyan, K. E.; Hardianto, G. The Effect of Platelet-Rich Plasma (PRP) Injection on Wound Healing and Sphincter Ani Muscle Tone in Post-Repair of Grade III-IV Perineal Tear Patients. *J. Med. Pharm. Chem. Res.* **2025**, *7* (8), 1655–1665.
(33) Romero-Cullerés, G.; Amela-Arévalo, A.; Jané-Feixas, C.; Vilaseca-Grané, A.; Arnau, A.; Torà, N. [Efficacy of collagen infiltrations in the pelvic pain caused by episiotomy and caesarean scars. Pilot randomized clinical trial]. *Rehabilitacion* **2022**, *56* (2), 85–92. https://doi.org/10.1016/j.rh.2021.04.001.
(34) Dasrilsyah, R. A.; Kalok ,Aida; Ng ,Beng Kwang; Ali ,Anizah; Teik Chew ,Kah; and Lim, P. S. Perineal Skin Tear Repair Following Vaginal Birth; Skin Adhesive versus Conventional Suture – a Randomised Controlled Trial. *J. Obstet. Gynaecol.* **2021**, *41* (2), 242–247. https://doi.org/10.1080/01443615.2020.1740917.
(35) Martino, C.; Salzano, F.; Martino, D.; Ralli, M.; De Vincentiis, M.; Maranzano, M.; Greco, A.; Salzano, G.; Di Stadio, A. A Prospective Randomized Trial of N-Butyl-Cyanoacrylate + Metacryloxysulfolane Adhesive versus Suture Alone for Grafting in Rhinoplasty: 9 Year Follow-Up. *Ann. Otol. Rhinol. Laryngol.* **2021**, *130* (5), 483–489. https://doi.org/10.1177/0003489420943910.
(36) Atesli, E. E.; Guven, S.; Senocak, G. N. C.; Guven, E. S. G. Comparison of the Aesthetic and Functional Efficacy of Subcuticular Running Closure (3/0 Rapid Absorbable 910 Polyglactin) with N-BUTYL Cyanoacrylate in Episiotomy Repair. *CEOG*, 2020, *47*, 660–663.

(37) Ochiai, A. M.; Araújo, N. M.; Moraes, S. D. T. A.; Caroci-Becker, A.; Sparvoli, L. G.; Teixeira, T. T.; Carvalho, R. R. The Use of Non-Surgical Glue to Repair Perineal First-Degree Lacerations in Normal Birth: A Non-Inferiority Randomised Trial. *Women Birth J. Aust. Coll. Midwives* **2021**, *34* (5), e514–e519. https://doi.org/10.1016/j.wombi.2020.09.018.
(38) Teixeira, T. T.; Riesco, M. L. Surgical Adhesive Glue to Repair First-Degree Perineal Tears in Vaginal Birth: A Randomised Controlled Clinical Trial. *Int. J. Nurs. Stud. Adv.* **2023**, *5*, 100130. https://doi.org/10.1016/j.ijnsa.2023.100130.
(39) Caroci-Becker, A.; Brunelli, W. S.; de Oliveira Pimentel Lima, M.; Ochiai, A. M.; Oliveira, S. G.; Riesco, M. L. Use of Surgical Glue versus Suture to Repair Perineal Tears: A Randomised Controlled Trial. *BMC Pregnancy Childbirth* **2023**, *23* (1), 246. https://doi.org/10.1186/s12884-023-05565-x.
(40) Feigenberg, T.; Maor-Sagie, E.; Zivi, E.; Abu-Dia, M.; Ben-Meir, A.; Sela, H. Y.; Ezra, Y. Using Adhesive Glue to Repair First Degree Perineal Tears: A Prospective Randomized Controlled Trial. *BioMed Res. Int.* **2014**, *2014* (1), 526590. https://doi.org/10.1155/2014/526590.
(41) Brunelli, W. S.; Caroci Becker, A.; Lima, M. O. P.; Oliveira, S. G.; Ochiai, A. M.; Caroci, L.; Araújo, N. M. D.; Riesco, M. L. Repercussions of Perineal Repair Using Surgical Glue or Suture Thread on Postpartum Outcomes: A Controlled Randomized Clinical Trial in São Paulo, Brazil. *Eur J Midwifery* **2024**, *8* (August), 1–11. https://doi.org/10.18332/ejm/191248.
(42) Chamariya, S.; Prasad, M.; Chauhan, A. Comparison of Dermabond Adhesive Glue with Skin Suture for Repair of Episiotomy. *Int. J. Reprod. Contracept. Obstet. Gynecol.* **2016**, *5* (10), 3461–3465. https://doi.org/10.18203/2320-1770.ijrcog20163423.
(43) Raya-Rivera, A. M.; Esquiliano, D.; Fierro-Pastrana, R.; López-Bayghen, E.; Valencia, P.; Ordorica-Flores, R.; Soker, S.; Yoo, J. J.; Atala, A. Tissue-Engineered Autologous Vaginal Organs in Patients: A Pilot Cohort Study. *The Lancet* **2014**, *384* (9940), 329–336. https://doi.org/10.1016/S0140-6736(14)60542-0.
(44) Cai, H.; Qiao, L.; Song, K.; He, Y. Oxidized, Regenerated Cellulose Adhesion Barrier Plus Intrauterine Device Prevents Recurrence After Adhesiolysis for Moderate to Severe Intrauterine Adhesions. *J. Minim. Invasive Gynecol.* **2017**, *24* (1), 80–88. https://doi.org/10.1016/j.jmig.2016.09.021.
(45) Zheng, F.; Xin, X.; He, F.; Liu, J.; Cui, Y. Meta-analysis on the Use of Hyaluronic Acid Gel to Prevent Intrauterine Adhesion after Intrauterine Operations. *Exp. Ther. Med.* **2020**, *19* (4), 2672–2678. https://doi.org/10.3892/etm.2020.8483.
(46) Fan, J.; Xie, J.; Liao, Y.; Lai, B.; Zhou, G.; Lian, W.; Xiong, J. Human Umbilical Cord-Derived Mesenchymal Stem Cells and Auto-Crosslinked Hyaluronic Acid Gel Complex for Treatment of Intrauterine Adhesion. *Aging* **2024**, *16* (7), 6273–6289. https://doi.org/10.18632/aging.205704.
(47) Hou, Z.; Yang, T.; Xu, D.; Fu, J.; Tang, H.; Zhao, J.; Zhang, Q.; Chen, J.; Qin, Q.; Li, W.; Chen, H.; Li, H.; Guo, L.; Xu, B.; Li, Y. hUC-MSCs Loaded Collagen Scaffold for Refractory Thin Endometrium Caused by Asherman Syndrome: A Double-Blind Randomized Controlled Trial. *Stem Cells Transl. Med.* **2025**, *14* (4). https://doi.org/10.1093/stcltm/szaf011.
(48) Cao, Y.; Sun, H.; Zhu, H.; Zhu, X.; Tang, X.; Yan, G.; Wang, J.; Bai, D.; Wang, J.; Wang, L.; Zhou, Q.; Wang, H.; Dai, C.; Ding, L.; Xu, B.; Zhou, Y.; Hao, J.; Dai, J.; Hu, Y. Allogeneic Cell Therapy Using Umbilical Cord MSCs on Collagen Scaffolds for Patients with Recurrent Uterine Adhesion: A Phase I Clinical Trial. *Stem Cell Res. Ther.* **2018**, *9* (1), 192. https://doi.org/10.1186/s13287-018-0904-3.
(49) Huang, J.; Li, Q.; Yuan, X.; Liu, Q.; Zhang, W.; Li, P. Intrauterine Infusion of Clinically Graded Human Umbilical Cord-Derived Mesenchymal Stem Cells for the Treatment of Poor Healing after Uterine Injury: A Phase I Clinical Trial. *Stem Cell Res. Ther.* **2022**, *13* (1), 85. https://doi.org/10.1186/s13287-022-02756-9.

(50) Zhang, Y.; Shi, L.; Lin, X.; Zhou, F.; Xin, L.; Xu, W.; Yu, H.; Li, J.; Pan, M.; Pan, Y.; Dai, Y.; Zhang, Y.; Shen, J.; Zhao, L.; Lu, M.; Zhang, S. Unresponsive Thin Endometrium Caused by Asherman Syndrome Treated with Umbilical Cord Mesenchymal Stem Cells on Collagen Scaffolds: A Pilot Study. *Stem Cell Res. Ther.* **2021**, *12* (1), 420. https://doi.org/10.1186/s13287-021-02499-z.
(51) Li, C.; Cai, A.; Sun, C.; Wu, B.; Chen, X.; Mao, Y.; Zhang, Y.; Gou, Y.; Yu, J.; Wang, Y.; Yu, H.; Wang, J. The Study on the Safety and Efficacy of Amnion Graft for Preventing the Recurrence of Moderate to Severe Intrauterine Adhesions. *Genes Dis.* **2020**, *7* (2), 266–271. https://doi.org/10.1016/j.gendis.2019.03.003.
(52) Amer, M. I.; Abd-El-Maeboud, K. H. I.; Abdelfatah, I.; Salama, F. A.; Abdallah, A. S. Human Amnion as a Temporary Biologic Barrier after Hysteroscopic Lysis of Severe Intrauterine Adhesions: Pilot Study. *J. Minim. Invasive Gynecol.* **2010**, *17* (5), 605–611. https://doi.org/10.1016/j.jmig.2010.03.019.
(53) Peng, X.; Li, T.; Zhao, Y.; Guo, Y.; Xia, E. Safety and Efficacy of Amnion Graft in Preventing Reformation of Intrauterine Adhesions. *J. Minim. Invasive Gynecol.* **2017**, *24* (7), 1204–1210. https://doi.org/10.1016/j.jmig.2017.08.005.
(54) Amer, M. I.; Abd-El-Maeboud, K. H. I.; Abdelfatah, I.; Salama, F. A.; Abdallah, A. S. Human Amnion as a Temporary Biologic Barrier after Hysteroscopic Lysis of Severe Intrauterine Adhesions: Pilot Study. *J. Minim. Invasive Gynecol.* **2010**, *17* (5), 605–611. https://doi.org/10.1016/j.jmig.2010.03.019.
(55) Amer, M. I.; Abd-El-Maeboud, K. H. Amnion Graft Following Hysteroscopic Lysis of Intrauterine Adhesions. *J. Obstet. Gynaecol. Res.* **2006**, *32* (6), 559–566. https://doi.org/10.1111/j.1447-0756.2006.00454.x.
(56) Azumaguchi, A.; Henmi, H.; Saito, T. Efficacy of Silicone Sheet as a Personalized Barrier for Preventing Adhesion Reformation after Hysteroscopic Adhesiolysis of Intrauterine Adhesions. *Reprod. Med. Biol.* **2019**, *18* (4), 378–383. https://doi.org/10.1002/rmb2.12294.
(57) Feng, L.; Sun, Y.; Zhang, S.; Qian, Y.; Fang, S.; Yang, B.; Xu, L.; Li, J.; Niu, Y.; Zhang, S.; Zhang, L.; Chen, J. A Novel Intrauterine Estrogen-Releasing System for Preventing the Postoperative Recurrence of Intrauterine Adhesion: A Multicenter Randomized Controlled Study. *BMC Med.* **2024**, *22* (1), 395. https://doi.org/10.1186/s12916-024-03608-4.
(58) Miyagi, M.; Mekaru, K.; Oishi, S.; Urasoe, C.; Akamine, K.; Aoki, Y. Silicone Plate for the Prevention of Postoperative Adhesions in Patients with Asherman Syndrome. *Case Rep. Obstet. Gynecol.* **2019**, *2019* (1), 5420837. https://doi.org/10.1155/2019/5420837.
(59) Hurst, B. S. Permanent Implantation of Expanded Polytetrafluoroethylene Is Safe for Pelvic Surgery. United States Expanded Polytetrafluoroethylene Reproductive Surgery Study Group. *Hum. Reprod. Oxf. Engl.* **1999**, *14* (4), 925–927. https://doi.org/10.1093/humrep/14.4.925.
(60) An Expanded Polytetrafluoroethylene Barrier (Gore-Tex**Gore-Tex Surgical Membrane; W.L. Gore and Associates, Inc., Flagstaff, Arizona. Surgical Membrane) Reduces Post-Myomectomy Adhesion formation††Supported by a Grant from W. L. Gore & Associates, Inc., Flagstaff, Arizona. *Fertil. Steril.* **1995**, *63* (3), 491–493. https://doi.org/10.1016/S0015-0282(16)57414-7.
(61) Jana, S. K.; Chakravarty, B.; Chaudhury, K. Letrozole and Curcumin Loaded-PLGA Nanoparticles: A Therapeutic Strategy for Endometriosis. *J. Nanomedicine Biotherapeutic Discov.* **2014**, *4* (1), 1000123.
(62) Chaichian, S.; Mehdizadeh Kashi, A.; Tehermanesh, K.; Pirhajati Mahabadi, V.; Minaeian, S.; Eslahi, N. Effect of PLGA Nanoparticle-Mediated Delivery of miRNA 503 on The Apoptosis of Ovarian Endometriosis Cells. *Cell J.* **2022**, *24* (11), 697–704. https://doi.org/10.22074/cellj.2022.557554.1069.
(63) Jensen, J. T.; Lukkari-Lax, E.; Schulze, A.; Wahdan, Y.; Serrani, M.; Kroll, R. Contraceptive Efficacy and Safety of the 52-Mg Levonorgestrel Intrauterine System for up to

8 Years: Findings from the Mirena Extension Trial. *Am. J. Obstet. Gynecol.* **2022**, *227* (6), 873.e1-873.e12. https://doi.org/10.1016/j.ajog.2022.09.007.
(64) Creinin, M. D.; Schreiber, C. A.; Turok, D. K.; Cwiak, C.; Chen, B. A.; Olariu, A. I. Levonorgestrel 52 Mg Intrauterine System Efficacy and Safety through 8 Years of Use. *Am. J. Obstet. Gynecol.* **2022**, *227* (6), 871.e1-871.e7. https://doi.org/10.1016/j.ajog.2022.05.022.
(65) Liu, P.; Meng, J.; Xiong, Y.; Wu, Y.; Xiao, Y.; Gao, S. Contraception with Levonorgestrel-Releasing Intrauterine System versus Copper Intrauterine Device: A Meta-Analysis of Randomized Controlled Trials. *eClinicalMedicine* **2024**, *78*. https://doi.org/10.1016/j.eclinm.2024.102926.
(66) Creinin, M. D.; Barnhart, K. T.; Gawron, L. M.; Eisenberg, D.; Mabey, R. G. J.; Jensen, J. T. Heavy Menstrual Bleeding Treatment With a Levonorgestrel 52-Mg Intrauterine Device. *Obstet. Gynecol.* **2023**, *141* (5), 971–978. https://doi.org/10.1097/AOG.0000000000005137.
(67) Huijs, D. P. C.; Derickx, A. J. M.; Beelen, P.; Leemans, J. C.; van Kuijk, S. M. J.; Bongers, M. Y.; Geomini, P. M. A. J. A 52-Mg Levonorgestrel-Releasing Intrauterine System vs Bipolar Radiofrequency Nonresectoscopic Endometrial Ablation in Women with Heavy Menstrual Bleeding: Long-Term Follow-up of a Multicenter Randomized Controlled Trial. *Am. J. Obstet. Gynecol.* **2024**, *230* (5), 542.e1-542.e10. https://doi.org/10.1016/j.ajog.2024.01.016.
(68) Croxatto, H. B.; Urbancsek, J.; Massai, R.; Bennink, H. C.; van Beek, A.; the Implanon® Study Group*. A Multicentre Efficacy and Safety Study of the Single Contraceptive Implant Implanon ®. *Hum. Reprod.* **1999**, *14* (4), 976–981. https://doi.org/10.1093/humrep/14.4.976.
(69) Darney, P.; Patel, A.; Rosen, K.; Shapiro, L. S.; Kaunitz, A. M. Safety and Efficacy of a Single-Rod Etonogestrel Implant (Implanon): Results from 11 International Clinical Trials. *Fertil. Steril.* **2009**, *91* (5), 1646–1653. https://doi.org/10.1016/j.fertnstert.2008.02.140.
(70) Bahamondes, L.; Bahamondes, M. V.; Juliato, C. R. T. Subdermal Contraceptive Implants. *Best Pract. Res. Clin. Obstet. Gynaecol.* **2025**, *100*, 102604. https://doi.org/10.1016/j.bpobgyn.2025.102604.
(71) Moray, K. V.; Chaurasia, H.; Sachin, O.; Joshi, B. A Systematic Review on Clinical Effectiveness, Side-Effect Profile and Meta-Analysis on Continuation Rate of Etonogestrel Contraceptive Implant. *Reprod. Health* **2021**, *18* (1), 4. https://doi.org/10.1186/s12978-020-01054-y.
(72) Dugré, N.; Choksi, N.; Kirkwood, J. Etonogestrel Implant Effectiveness. *Can. Fam. Physician* **2022**, *68* (8), 594. https://doi.org/10.46747/cfp.6808594.
(73) Weisberg, E.; Hickey, M.; Palmer, D.; O'Connor, V.; Salamonsen, L. A.; Findlay, J. K.; Fraser, I. S. A Randomized Controlled Trial of Treatment Options for Troublesome Uterine Bleeding in Implanon Users. *Hum. Reprod. Oxf. Engl.* **2009**, *24* (8), 1852–1861. https://doi.org/10.1093/humrep/dep081.
(74) Oddsson, K.; Leifels-Fischer, B.; de Melo, N. R.; Wiel-Masson, D.; Benedetto, C.; Verhoeven, C. H. J.; Dieben, T. O. M. Efficacy and Safety of a Contraceptive Vaginal Ring (NuvaRing) Compared with a Combined Oral Contraceptive: A 1-Year Randomized Trial. *Contraception* **2005**, *71* (3), 176–182. https://doi.org/10.1016/j.contraception.2004.09.001.
(75) Gruber, C. J. The Combined Contraceptive Vaginal Ring (NuvaRing): Evaluation of the Clinical and Pharmacological Evidence. *Womens Health* **2006**, *2* (3), 351–356. https://doi.org/10.2217/17455057.2.3.351.
(76) Selvan, P.; Piran, P.; Balucani, C.; Tark, B.; Adler, Z.; Levine, S. R. Stroke and Etonogestrel/Ethinyl Estradiol Ring (NuvaRing): Clinical, Radiological, and Prognostic Features. *J. Stroke Cerebrovasc. Dis.* **2017**, *26* (3), 608–617. https://doi.org/10.1016/j.jstrokecerebrovasdis.2016.11.111.
(77) Nanah, A.; Daw, H.; Abdelghaffar, B. Venous Thromboembolic Disease Provoked by Hormone-Eluting Vaginal Rings. *Am. J. Ther.* **2024**, *31* (4).

(78) Creinin, M. D.; Gawron, L. M.; Roe, A. H.; Blumenthal, P. D.; Boraas, C. M.; Hou, M. Y.; McNicholas, C.; Schreifels, M. J.; Peters, K.; Culwell, K.; Turok, D. K. Three-Year Efficacy, Safety, and Tolerability Outcomes from a Phase 3 Study of a Low-Dose Copper Intrauterine Device. *Contraception* **2025**, *143*, 110771. https://doi.org/10.1016/j.contraception.2024.110771.
(79) Kaneshiro, B.; Aeby, T. Long-Term Safety, Efficacy, and Patient Acceptability of the Intrauterine Copper T-380A Contraceptive Device. *Int. J. Womens Health* **2010**, *2*, 211–220. https://doi.org/10.2147/ijwh.s6914.
(80) Perelló-Capó, J.; Estadella-Tarriel, J.; Gich-Saladich, I.; Bailón-Queiruga, M.; Llurba-Olivé, E.; Calaf-Alsina, J. Health-Related Quality of Life and Satisfaction Associated with 3-Year Use of a Levonorgestrel 13.5 Mg Intrauterine Device vs Nova T Copper 380 Mm2 Intrauterine Device: Results of a Phase 4 Randomized Controlled Trial. *Contraception* **2024**, *133*, 110367. https://doi.org/10.1016/j.contraception.2024.110367.
(81) Turok, D. K.; Nelson, A. L.; Dart, C.; Schreiber, C. A.; Peters, K.; Schreifels, M. J.; Katz, B. Efficacy, Safety, and Tolerability of a New Low-Dose Copper and Nitinol Intrauterine Device: Phase 2 Data to 36 Months. *Obstet. Gynecol.* **2020**, *135* (4), 840–847. https://doi.org/10.1097/AOG.0000000000003756.
(82) van Gastel, D. M.; Maassen, L. W.; Koks, C. A. M.; Veersema, S.; Bongers, M. Y. Challenges in Removing the Essure® Device. *Case Rep. Obstet. Gynecol.* **2020**, *2020* (1), 8823606. https://doi.org/10.1155/2020/8823606.
(83) Sharma, R. S.; Mathur, A. K.; Das, H. C.; Shah, B. S.; Goyal, A.; Sharma, K. C.; Abrol, S.; Sahoo, B.; Lohiya, N. K.; Sadasukhi, T. C. Phase-III Clinical Trial with an Intravasal Once Injectable Non-Hormonal Male Contraceptive-Reversible Inhibition of Sperm under Guidance (RISUG). *Androl.-Open Access* **2023**, *12* (5), 1000297.
(84) Lohiya, N. K.; Ansari, A. S.; Sadasukhi, T. C.; Pachera, S.; Khilwani, B.; Dhaked, R. K. RISUG® Offers Early Contraception: An Experience during Phase III Clinical Trials. *J. Reprod. Healthc. Med.* **2022**, *3* (11).
(85) Sharma, R. S.; Mathur, A. K.; Singh, R.; Das, H. C.; Singh, G. J.; Toor, D. P. S.; Guha, S. K. Safety & Efficacy of an Intravasal, One-Time Injectable & Non-Hormonal Male Contraceptive (RISUG): A Clinical Experience. *Indian J. Med. Res.* **2019**, *150* (1).
(86) Singh, A. K. Intra-Vas Deferens Injection of Styrene Maleic Anhydride Gel for Male Contraception: Is It Safe? *J. Fam. Plann. Reprod. Health Care* **2002**, *28* (4), 208. https://doi.org/10.1783/147118902101196603.
(87) Nakagawa, K.; Horikawa, T.; Orita, Y.; Yamashiro, E.; Watanabe, H.; Shirai, A.; Ogata, S.; Kataoka, H.; Kuroda, K.; Takamizawa, S.; Sugiyama, R. Hyaluronan-Enriched Transfer Medium (HETM) Can Improve the Implantation Rate in Morphologically Poor Euploid Blastocyst Transfer. *Arch. Gynecol. Obstet.* **2023**, *308* (2), 611–619. https://doi.org/10.1007/s00404-023-07083-9.
(88) Bhoi, N. R.; Murdia, N.; Murdia, K.; Chandra, V.; Suwalka, I.; Mistari, W.; Aggrawal, R.; Shah, N.; Kumar, D. Effect of Hyaluronic Acid-Containing Transfer Media (EmbryoGlue®) on the Live Birth Rate in Frozen Thawed Embryo Transfer Cycles. *Cureus* **2024**, *16* (1), e52713. https://doi.org/10.7759/cureus.52713.
(89) Fancsovits, P.; Lehner, A.; Murber, A.; Kaszas, Z.; Rigo, J.; Urbancsek, J. Effect of Hyaluronan-Enriched Embryo Transfer Medium on IVF Outcome: A Prospective Randomized Clinical Trial. *Arch. Gynecol. Obstet.* **2015**, *291* (5), 1173–1179. https://doi.org/10.1007/s00404-014-3541-9.
(90) Adeniyi, T.; Horne, G.; Ruane, P. T.; Brison, D. R.; Roberts, S. A. Clinical Efficacy of Hyaluronate-Containing Embryo Transfer Medium in IVF/ICSI Treatment Cycles: A Cohort Study. *Hum. Reprod. Open* **2021**, *2021* (1), hoab004. https://doi.org/10.1093/hropen/hoab004.

(91) Sellers, R.; Ten, J.; Arnedo, A. R.; Guerrero, J.; Moliner, B.; Llácer, J.; Bernabeu, R. The Use of Embryoglue® (EG) for Embryo Transfer Does Not Improve Live Birth Rates: A Prospective Randomized Controlled Trial. *Reprod. Biomed. Online* **2022**, *45*, e47. https://doi.org/10.1016/j.rbmo.2022.08.081.
(92) Child, T. J.; Bevan, A.; Frettsome-Hook, R.; Craig, J.; Shahbazian, S.; Mounce, G. A RANDOMISED CONTROLLED BLINDED TRIAL ASSESSING THE EFFECTIVENESS OF EMBRYOGLUE AS AN EMBRYO TRANSFER MEDIUM IN IVF CYCLES. *Fertil. Steril.* **2021**, *116* (3), e4. https://doi.org/10.1016/j.fertnstert.2021.07.019.
(93) Balakier, H.; Kuznyetsova ,Iryna; and Librach, C. L. The Impact of Hyaluronan-Enriched Culture Medium and Intrauterine Infusion of Human Chorionic Gonadotropin on Clinical Outcomes in Blastocyst Transfer Cycles. *Syst. Biol. Reprod. Med.* **2020**, *66* (2), 79–88. https://doi.org/10.1080/19396368.2020.1727995.
(94) Korosec, S.; Virant-Klun, I.; Tomazevic, T.; Zech, N. H.; Meden-Vrtovec, H. Single Fresh and Frozen–Thawed Blastocyst Transfer Using Hyaluronan-Rich Transfer Medium. *Reprod. Biomed. Online* **2007**, *15* (6), 701–707. https://doi.org/10.1016/S1472-6483(10)60538-X.
(95) Heymann, D.; Vidal, L.; Shoham, Z.; Kostova, E.; Showell, M.; Or, Y. The Effect of Hyaluronic Acid in Embryo Transfer Media in Donor Oocyte Cycles and Autologous Oocyte Cycles: A Systematic Review and Meta-Analysis. *Hum. Reprod. Oxf. Engl.* **2022**, *37* (7), 1451–1469. https://doi.org/10.1093/humrep/deac097.
(96) Laronda, M. M.; Rutz, A. L.; Xiao, S.; Whelan, K. A.; Duncan, F. E.; Roth, E. W.; Woodruff, T. K.; Shah, R. N. A Bioprosthetic Ovary Created Using 3D Printed Microporous Scaffolds Restores Ovarian Function in Sterilized Mice. *Nat. Commun.* **2017**, *8* (1), 15261. https://doi.org/10.1038/ncomms15261.
(97) Wu, T.; Gao, Y.-Y.; Tang, X.-N.; Zhang, J.-J.; Wang, S.-X. Construction of Artificial Ovaries with Decellularized Porcine Scaffold and Its Elicited Immune Response after Xenotransplantation in Mice. *J. Funct. Biomater.* **2022**, *13* (4). https://doi.org/10.3390/jfb13040165.
(98) Buckenmeyer, M. J.; Sukhwani, M.; Iftikhar, A.; Nolfi, A. L.; Xian, Z.; Dadi, S.; Case, Z. W.; Steimer, S. R.; D'Amore, A.; Orwig, K. E.; Brown, B. N. A Bioengineered in Situ Ovary (ISO) Supports Follicle Engraftment and Live-Births Post-Chemotherapy. *J. Tissue Eng.* **2023**, *14*, 20417314231197282. https://doi.org/10.1177/20417314231197282.
(99) Wu, T.; Huang, K.-C.; Yan, J.-F.; Zhang, J.-J.; Wang, S.-X. Extracellular Matrix-Derived Scaffolds in Constructing Artificial Ovaries for Ovarian Failure: A Systematic Methodological Review. *Hum. Reprod. Open* **2023**, *2023* (2), hoad014. https://doi.org/10.1093/hropen/hoad014.
(100) Vanacker, J.; Luyckx, V.; Dolmans, M.-M.; Des Rieux, A.; Jaeger, J.; Van Langendonckt, A.; Donnez, J.; Amorim, C. A. Transplantation of an Alginate–Matrigel Matrix Containing Isolated Ovarian Cells: First Step in Developing a Biodegradable Scaffold to Transplant Isolated Preantral Follicles and Ovarian Cells. *Biomaterials* **2012**, *33* (26), 6079–6085. https://doi.org/10.1016/j.biomaterials.2012.05.015.
(101) Laronda, M. M.; Duncan, F. E.; Hornick, J. E.; Xu, M.; Pahnke, J. E.; Whelan, K. A.; Shea, L. D.; Woodruff, T. K. Alginate Encapsulation Supports the Growth and Differentiation of Human Primordial Follicles within Ovarian Cortical Tissue. *J. Assist. Reprod. Genet.* **2014**, *31* (8), 1013–1028. https://doi.org/10.1007/s10815-014-0252-x.
(102) Vanacker, J.; Amorim, C. A. Alginate: A Versatile Biomaterial to Encapsulate Isolated Ovarian Follicles. *Ann. Biomed. Eng.* **2017**, *45* (7), 1633–1649. https://doi.org/10.1007/s10439-017-1816-6.
(103) Feyzmanesh, S.; Halvaei, I.; Baheiraei, N. Alginate Effects on Human Sperm Parameters during Freezing and Thawing: A Prospective Study. *Cell J.* **2022**, *24* (7), 417–423. https://doi.org/10.22074/cellj.2022.8122.

(104) Pirnia, A.; Parivar, K.; Hemadi, M.; Yaghmaei, P.; Gholami, M. Stemness of Spermatogonial Stem Cells Encapsulated in Alginate Hydrogel during Cryopreservation. *Andrologia* **2017**, *49* (5), e12650. https://doi.org/10.1111/and.12650.
(105) Abdul-Al, M.; Zaernia, A.; Sefat, F. Biomaterials for Breast Reconstruction: Promises, Advances, and Challenges. *J. Tissue Eng. Regen. Med.* **2020**, *14* (11), 1549–1569. https://doi.org/10.1002/term.3121.
(106) Berkane, Y.; Oubari, H.; van Dieren, L.; Charlès, L.; Lupon, E.; McCarthy, M.; Cetrulo, C. L. J.; Bertheuil, N.; Uygun, B. E.; Smadja, D. M.; Lellouch, A. G. Tissue Engineering Strategies for Breast Reconstruction: A Literature Review of Current Advances and Future Directions. *Ann. Transl. Med.* **2024**, *12* (1), 15. https://doi.org/10.21037/atm-23-1724.
(107) Park, J.-H.; Park, J.-U.; Chang, H. Advances in Biomaterials for Breast Reconstruction. *Appl. Sci.* **2021**, *11* (16). https://doi.org/10.3390/app11167493.
(108) Lv, X.; Xiang, C.; Zheng, Y.; Xu, S.-F.; Zhou, W.-X.; Lv, X.-L. A Review: Recent Advances in the Application of 3D Printing Biomaterials in Breast Reconstruction Research. *Eur. J. Plast. Surg.* **2024**, *47* (1), 38. https://doi.org/10.1007/s00238-024-02171-8.
